# Perinatal outcomes of neonates born from different endometrial preparation protocols after frozen embryo transfer: a retrospective cohort study

**DOI:** 10.1186/s12884-021-03791-9

**Published:** 2021-04-29

**Authors:** Cheng Li, Yi-Chen He, Jing-Jing Xu, Yu Wang, Han Liu, Chen-Chi Duan, Chao-Yi Shi, Lei Chen, Jie Wang, Jian-Zhong Sheng, He-Feng Huang, Yan-Ting Wu

**Affiliations:** 1 International Peace Maternity and Child Health Hospital, School of Medicine, Shanghai Jiao Tong University, No.910, Hengshan Rd., Shanghai, 200030 China; 2 Shanghai Key Laboratory of Embryo Original Diseases, Shanghai, 200030 China; 3 Department of Pathology and Pathophysiology, School of Medicine, Zhejiang University, Hangzhou, Zhejiang China; 4 Obstetrics and Gynecology Hospital, Fudan University, No.419, Fangxie Rd., Shanghai, 200000 China

**Keywords:** Frozen-thawed embryo transfer, Endometrium preparation, Gestational hypertensive disorder, Intrahepatic cholestasis of pregnancy, Small for gestational age

## Abstract

**Background:**

Previous studies have focused on pregnancy outcomes after frozen embryo transfer (FET) performed using different endometrial preparation protocols. Few studies have evaluated the effect of endometrial preparation on pregnancy-related complications. This study was designed to explore the association between different endometrial preparation protocols and adverse obstetric and perinatal complications after FET.

**Methods:**

We retrospectively included all FET cycles (*n* = 12,950) in our hospital between 2010 and 2017, and categorized them into three groups, natural cycles (NC), hormone replacement therapy (HRT) and ovarian stimulation (OS) protocols. Pregnancy-related complications and subsequent neonatal outcomes were compared among groups.

**Results:**

Among all 12,950 FET cycles, the live birth rate was slightly lower for HRT cycles than for NC (HRT vs. NC: 28.15% vs. 31.16%, *p* < 0.001). The pregnancy loss rate was significantly higher in OS or HRT cycles than in NC (HRT vs. NC: 17.14% vs. 10.89%, *p* < 0.001; OS vs. NC: 16.44% vs. 10.89%, *p* = 0.001). Among 3864 women with live birth, preparing the endometrium using OS or HRT protocols increased the risk of preeclampsia, and intrahepatic cholestasis of pregnancy (ICP) in both singleton and multiple deliveries. Additionally, OS and HRT protocols increased the risk of low birth weight (LBW) and small for gestational age (SGA) in both singletons and multiples after FET.

**Conclusion:**

Compared with HRT or OS protocols, preparing the endometrium with NC was associated with the decreased risk of pregnancy-related complications, as well as the decreased risk of LBW and SGA after FET.

**Supplementary Information:**

The online version contains supplementary material available at 10.1186/s12884-021-03791-9.

## Background

Frozen-thawed embryo transfer (FET) has been increasingly used in assisted reproductive technology (ART) to avoid the inferior effects of fresh embryo transfer, which include high E2 levels, and to surplus embryos after oocyte retrieval and in vitro fertilization (IVF) to improve cumulative pregnancy rates [1, 2]. As laboratory techniques have improved, especially those related to the enhanced survival and implantation rates achieved by vitrification, the number of FET cycles performed has increased dramatically over the last decade [3].

The synchronization of embryo and endometrial development is regarded as a crucial factor in the success of FET. In contrast to the complicated controlled ovarian hyperstimulation protocols employed to stimulate follicular growth for IVF, it is much simpler to prepare the endometrium with the aim of improving its receptivity. Endometrial preparation protocols can be commonly divided into three methods: natural cycles (NC) using spontaneous ovulation, ovarian stimulation (OS) protocols performed with endogenous steroids, and hormone replacement therapy (HRT) protocols involving artificial preparation with exogenous steroids [4, 5]. Many cohorts have been studied and randomized controlled trials performed to evaluate the effects of these endometrial preparation protocols on pregnancy outcomes for FET; however, their findings remain controversial [5–8]. Although preparing the endometrium with exogenous hormones provides great advantages, such as minimizing monitoring and scheduling the timing of the procedure, the abnormal hormone levels observed during the window of implantation and the dysfunction of hormone response elements on endometrium induced by the HRT or OS protocols should not be ignored [9–11]. Our previous study has reported that the risk of adverse obstetric and neonatal outcomes after FET is increased by ovarian stimulation-induced superphysiological estradiol levels, potentially influencing the future health of offspring produced using this technique [12]. Compared to the pregnancy outcomes achieved after FET, few studies have explored the effect of endometrial preparation on pregnancy-related complications and subsequent neonatal outcomes.

Understanding the effects of different endometrial preparation protocols on pregnancy-related complications and perinatal outcomes can further optimize maternal and child health after FET. Therefore, this cohort study was designed to explore the associations between maternal exposure to different endometrial preparation protocols and perinatal outcomes, including pregnancy rates, adverse obstetric complications, and neonatal outcomes.

## Methods

### Study design and participants

We retrospectively recruited all infertile women undergoing FET at International Peace Maternity and Child Health Hospital from May 2010 to September 2017. Women who underwent preimplantation genetic testing (PGT). All Chinese participants were categorized into three groups according to the protocols of endometrial preparation (nature cycles, OS protocols, or HRT protocols). Participants with live birth deliveries were included in the perinatal outcome analysis.

All procedures and follow-up performed in this study involving human participants were conducted in accordance with the ethical standards of the Institutional Review Board of the International Peace Maternity and Child Health Hospital, Shanghai, China (GKLW-2016-21). The reporting of this study conforms to the STROBE statement.

### ART procedures

The process of ART was conducted as we previously described [12]. Briefly, infertile women undergo controlled ovarian hyperstimulation (COH), oocyte retrieval, and then insemination by either conventional in vitro fertilization (IVF) or intracytoplasmic sperm injection (ICSI). For women undergoing FET, embryos are cryopreserved via vitrification. Before embryo thawing, the endometrium was prepared by natural monitoring, HRT or an OS cycle.

Normally, NC was the favorable choice for women with regular menstruation. HRT or OS cycle was considered as preferred choice for women with irregular menstruation or history of anovulation. For women underwent NC, no medication was administrated during the follicular phase. For women underwent HRT cycles, valerate estrogen was administered orally until the endometrial thickness reached up to 7 mm, dydrogesterone was administered orally, together with progesterone was administered vaginally for luteal phase support. For women underwent OS protocols, human menopausal gonadotropin was started on day 3–5, dose of HMG was adjusted according to the follicles diameters and the levels of serum hormone steroids. Urinary hCG was administrated at when follicles diameters reached at least 18 mm.

Serum hormone levels were assessed 3–5 days before embryo transfer, including E2, progesterone (P4) and luteinizing hormone (LH), in the hospital clinical chemistry laboratory. Endometrial thickness was also measured before FET by highly trained sonographers via transvaginal ultrasound (Acuson X300, Siemens, Germany). In our hospital, all embryo transfer procedures were performed under transabdominal ultrasound guidance, despite transvaginal ultrasound guidance can improve the percentage of pregnancies per transfer [13, 14].

### Data collection and variable definition

All participants were interviewed in person to obtain information on sociodemographic characteristics (including maternal age at oocyte retrieval and embryo transfer, residence, educational level, occupation, and smoking status during pregnancy), reproductive history (including parity, previous abortions, previous ectopic pregnancy, cause of infertility, duration of infertility, and primary infertility). Before initiating IVF cycles, height and weight were measured for each patient, and the patient’s body mass index (BMI) was then calculated.

Details on the use of oocyte retrieval and frozen-thawed embryo transfer were abstracted from the patient’s hospital records as we previously described [15]. ART procedures were conducted using routine protocols, and information was documented, including the COH protocol, type of insemination, number of oocytes retrieved, the day of embryo transfer, and the number of embryos transferred.

Pregnancy-related complications were abstracted from the participants’ health records, including gestational hypertensive disorder, gestational diabetes mellitus (GDM), intrahepatic cholestasis of pregnancy (ICP), meconium staining of the amniotic fluid, preterm birth, and mode of delivery. The birth weight and sex of all neonates were also recorded. The weight for gestational age of each neonate was defined according to a global reference for birth weight for a given gestational age and sex [16].

All information on the sociodemographic characteristics, reproductive history, ARTs procedures, and the diagnosis of pregnancy-related complications, were exported from the electronic database by department of information.

### Statistical analysis

Continuous variables with normal distributions are represented as the means ± standard deviations, and differences were tested by one-way analysis of variance. Continuous variables with skewed distributions are shown as medians with interquartile ranges, and differences were tested by the Kruskal-Wallis test. Categorical outcome variables are represented as frequencies with proportions, and the Cochran-Mantel-Haenszel χ2 test was used to detect the differences. Multiple comparisons of pregnancy outcomes among groups were detected by multinomial logistic regression.

The outcomes of pregnancy-related complications as well as neonatal birth weight were assessed according to the stratification of singleton or multiple deliveries. To explore the associations between the different endometrial preparation protocols and adverse pregnancy-related complications for both singletons and multiple deliveries, odds ratios (ORs) and 95% confidence intervals (CIs) were calculated and adjusted for potential confounding factors for each outcome using multivariable logistic regression. The analysis of neonatal birth weight of singletons was performed by multinomial logistic regression analyses. The analysis of neonatal birth weight of multiples was performed via multilevel logistic regression according to Carlin et al. [17].

All statistical analyses were conducted using SAS software version 9.3 (SAS Institute, Inc., Cary, NC). *P* values less than 0.05 were considered statistically significant. For multiple comparisons, *P* values less than 0.017 were considered significantly different.

## Results

**Fig. 1:**
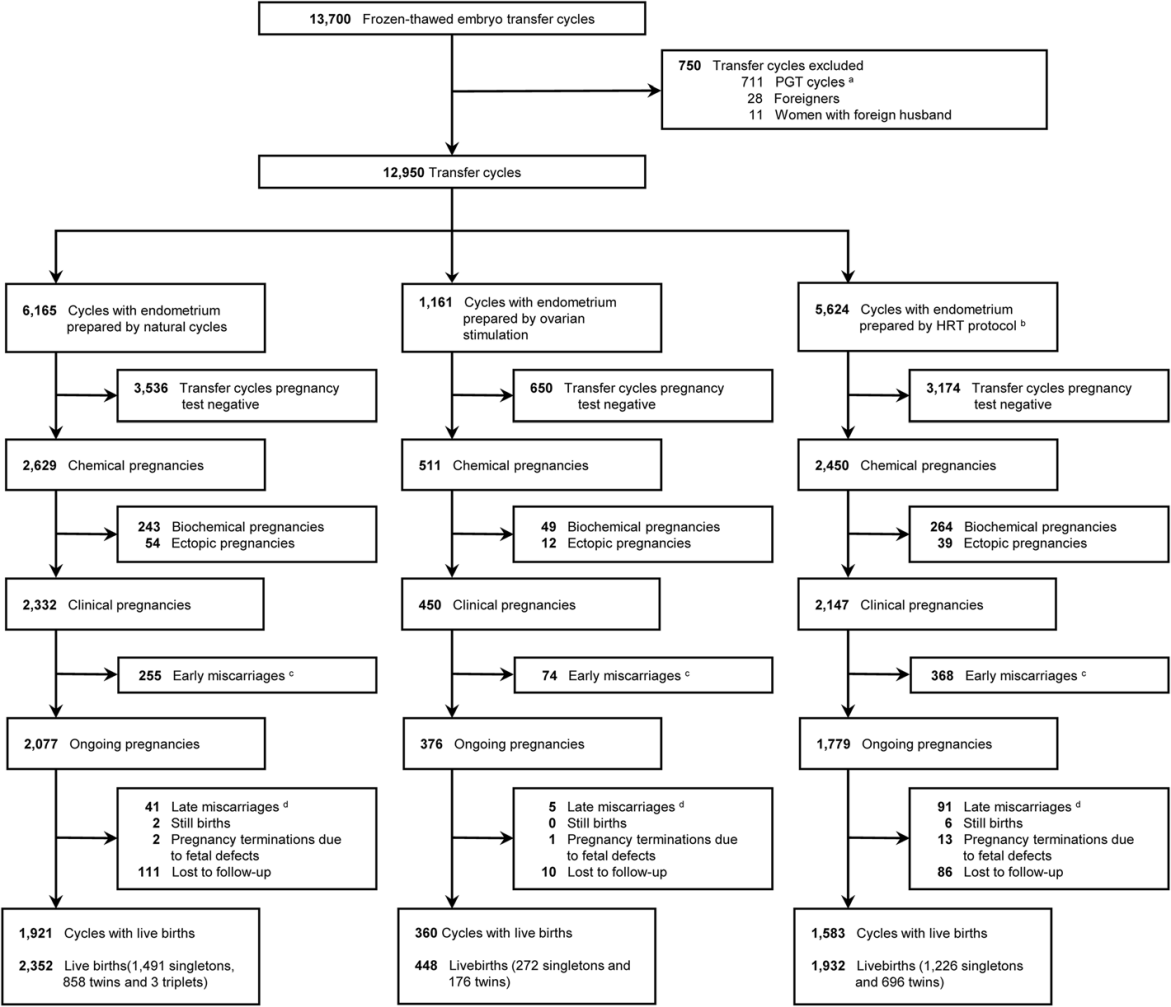
Study flow chart. **a** PGT, preimplantation genetic testing. **b** HRT, hormone replacement therapy. **c** Early miscarriage was defined as spontaneous loss of pregnancy before 12 gestational weeks. **d** Late miscarriage was defined as pregnancy loss between 12 and 28 gestational weeks

The flow of the study participants is shown in Fig. 1. A total of 12,950 FET cycles met the inclusion criteria of this study, and 4732 women with live birth deliveries (2997 singletons and 1735 multiples) were included in the analysis. In all, 207 participants were lost to follow-up.

**Fig. 2:**
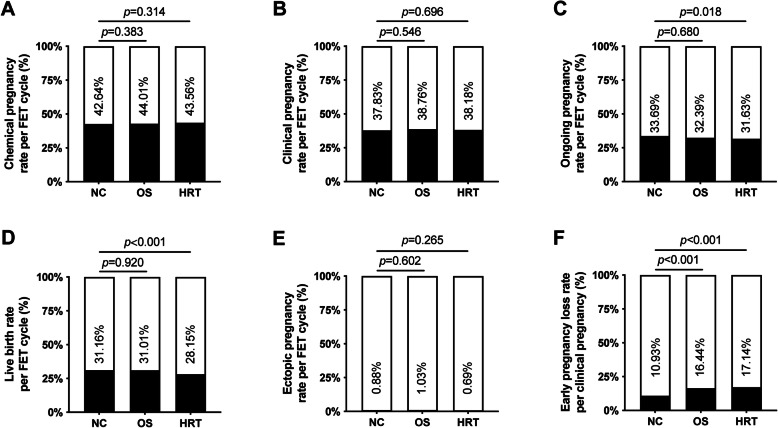
Chemical pregnancy rate (**a**), clinical pregnancy rate (**b**), ongoing pregnancy rate (**c**), live birth rate (**d**), ectopic pregnancy rate (**e**), and early pregnancy loss rate (**f**) in FET following different endometrial preparation protocols. FET, frozen-thawed embryo transfer; NC, natural cycles; OS, ovarian stimulation; HRT, hormone replacement therapy. Multiple comparisons of pregnancy outcomes among groups were performed by multinomial logistic regression. Chemical pregnancy was defined as an elevated serum β-hCG level of more than 10 mIU/ml. Chemical pregnancy rate was defined as the number of chemical pregnancy divided by the number of embryo transfer cycle for each group. Clinical pregnancy was defined as a pregnancy documented by ultrasound at 6–8 gestational weeks that showed a gestational sac inside the uterus. Clinical pregnancy rate was defined as the number of clinical pregnancy divided by the number of embryo transfer cycle for each group. Ongoing pregnancy was defined as a pregnancy documented by ultrasound at 12 gestational weeks that showed the presence of fetal heartbeat. Ongoing pregnancy rate was defined as the number of ongoing pregnancy divided by the number of embryo transfer cycle for each group. Live birth was defined as the delivery of one or more infants with any signs of life after 28 gestational weeks. Live birth rate (% per embryo transfer cycle) was defined as the number of live birth divided by the number of embryo transfer cycle for each group. Ectopic pregnancy was defined as an embryo implanted outside the uterine. Ectopic pregnancy rate was defined as the number of ectopic pregnancy divided by the number of embryo transfer cycle for each group. Early pregnancy loss was defined as a pregnancy loss before 12 gestational weeks. Early pregnancy loss rate was defined as the number of early pregnancy loss divided by the number of clinical pregnancy for each group

The pregnancy outcomes per transfer cycle of the three groups are shown in Fig. 2. There were no significant differences in the chemical pregnancy rate (NC vs. OS vs. HRT: 42.64% vs. 44.01% vs. 43.56%), clinical pregnancy rate (NC vs. OS vs. HRT: 37.83% vs. 38.76% vs. 38.18%) or ongoing pregnancy rate (NC vs. OS vs. HRT: 33.69% vs. 32.39% vs. 31.63%) among the three groups. However, the rate of live birth was lower in patients who underwent HRT cycles than in those who underwent an NC cycle (HRT vs. NC: 28.15% vs. 31.16%, *p* < 0.001), while a comparable live birth rate was observed between patients who underwent endometrium preparation with the NC or OS protocol (NC vs. OS: 31.16% vs. 31.01%, *p* = 0.920). Additionally, patients from not only the OS group (OS vs. NC: 16.44% vs. 10.89%, *p* = 0.001) but also the HRT group (HRT vs. NC: 17.14 vs. 10.89%, *p* < 0.001) had a higher early pregnancy loss rate than was observed in the NC group. The rate of ectopic pregnancy was similar among the three groups.

To analyze the associations among different endometrial preparation protocols in FET cycles and adverse pregnancy-related complications as well as perinatal outcomes, 4732 women with live birth deliveries were included in the analysis. Table 1 shows the distributions of maternal sociodemographic characteristics and reproductive histories among the groups. Notably, differences in maternal age at oocyte retrieval and embryo transfer were small and unlikely to be clinically significant. The causes of infertility were distributed differently among the groups. Anovulatory patients were more likely to undergo endometrium preparation with an OS or HRT protocol than the NC protocol (NC vs. OS vs. HRT: 1.25% vs. 8.33% vs. 11.36%, *p* < 0.001). However, patients with normal ovarian function, such as tubal infertility or male-factor infertility, were more likely to undergo endometrium preparation with the NC protocol (*p* < 0.001).

**Table 1:** Maternal characteristics of pregnancies carried to delivery following FET with different endometrial preparation protocols

	NC (***N*** = 1921)	OS (***N*** = 360)	HRT (***N*** = 1583)	***p***
	No. (%)	No. (%)	No. (%)	
**Maternal socio-demographic characteristics**				
**Age of oocyte retrieval, Mean ± SD, years**	31.25 ± 3.74	30.95 ± 3.81	30.73 ± 3.69	< 0.001
**Age of embryo transfer, Mean ± SD, years**	31.43 ± 3.74	31.04 ± 3.78	30.96 ± 3.70	< 0.001
**Pre-gestational BMI, Mean ± SD, kg/m**^**2**^	22.42 ± 3.02	22.55 ± 2.99	22.35 ± 3.24	0.216
**Residence**				
Residents	1271 (66.16)	228 (63.33)	1057 (66.77)	0.731
Immigrants	650 (33.84)	132 (36.67)	526 (33.23)	
**Education attainment**				
Primary school or lower	21 (1.09)	8 (2.22)	16 (1.01)	0.386
Middle school	223 (11.61)	43 (11.94)	207 (13.08)	
High school	316 (16.45)	57 (15.83)	271 (17.12)	
Collage or above	1361 (70.85)	252 (70.00)	1089 (69.79)	
**Occupation**				
Employed	1339 (69.70)	219 (60.83)	1127 (71.19)	0.735
Self-employed	400 (20.82)	108 (30.00)	297 (18.76)	
Unemployed	182 (9.47)	33 (9.17)	159 (10.04)	
**Smoking during pregnancy**				
No	1898 (98.80)	357 (99.17)	1571 (99.24)	0.187
Yes	23 (1.20)	3 (0.83)	12 (0.76)	
**History of reproduction**				
**Parity**				
No	1754 (91.83)	333 (92.50)	1475 (93.18)	0.132
Yes	157 (8.17)	27 (7.50)	108 (6.82)	
**Number of previous abortions**				
0	1298 (67.57)	227 (63.06)	1093 (69.05)	0.147
1–2	560 (29.15)	124 (34.44)	448 (28.30)	
≥ 3	63 (3.28)	9 (2.50)	42 (2.65)	
**Previous ectopic pregnancy**				
No	1668 (86.83)	316 (87.78)	1378 (87.05)	0.837
Yes	253 (13.17)	44 (12.22)	205 (12.95)	
**Duration of infertility**				
1–2	747 (38.89)	133 (36.94)	589 (37.21)	0.195
3–4	592 (30.82)	133 (36.94)	532 (33.61)	
≥ 5	582 (30.30)	94 (26.11)	462 (29.19)	
**Primary infertility**				
No	1104 (57.47)	207 (57.50)	924 (58.37)	0.954
Yes	817 (42.53)	153 (42.50)	659 (41.63)	
**Causes of infertility**				
Tubal infertility	924 (48.10)	155 (43.06)	654 (41.31)	< 0.001
Anovulatory	24 (1.25)	30 (8.33)	183 (11.56)	
Endometriosis	55 (2.86)	4 (1.11)	44 (2.78)	
Male-factor infertility	159 (8.28)	19 (5.28)	106 (6.70)	
Unexplained infertility	321 (16.71)	70 (19.44)	196 (12.38)	
Combined ^a^	438 (22.80)	82 (22.78)	400 (25.27)	

*FET* frozen embryo transfer, *NC* natural cycles, *OS* ovarian stimulation, *HRT* hormonal replacement therapy, *BMI* body mass index

^a^Combined was defined as two or more infertile causes mentioned above

Table 2 presents the distributions of the procedures used for oocyte retrieval and frozen-thawed embryo transfer among each group. The distribution of COH protocols (*p* = 0.345), type of insemination (*p* = 0.901) and number of oocytes retrieved (*p* = 0.171) were similar among the groups. With regard to FET procedures, no difference was found in the day of embryo transfer. (*p* = 0.724), number of embryo transferred (*p* = 0.811) or thickness of the endometrium (*p* = 0.952) among the three groups. However, the hormone levels obtained before FET were significantly different among the endometrial preparation protocols. The results showed that E2 levels were significantly higher in the OS and HRT groups than in the NC group (NC vs. OS vs. HRT: 1.20 [0.86–1.88] vs. 1.64 [1.01–2.69] vs. 1.86 [1.07–3.22] × 10^3^ pmol/L, *p* < 0.001). P4 levels were lower in the HRT group than in the OS and NC groups (NC vs. OS vs. HRT: 2.90 [1.80–3.60] vs. 2.50 [1.50–2.60] vs. 1.50 [0.90–2.40] nmol/L, *p* < 0.001). Additionally, LH levels were also different after endometrial preparation among the groups (NC vs. OS vs. HRT: 14.05 [9.20–24.90] vs. 10.60 [6.90–17.40] vs. 12.00 [7.50–20.30] IU/L, *p* < 0.001).

**Table 2:** ART procedures of pregnancies carried to delivery following FET with different endometrial preparation protocols

	NC (***N*** = 1921)	OS (***N*** = 360)	HRT (***N*** = 1583)	***p***
	No. (%)	No. (%)	No. (%)	
**Characteristics of oocytes retrieval cycle**				
**COH protocol**				
GnRH-agonist regimen	897 (46.69)	181 (50.28)	743 (46.94)	0.345
GnRH-antagonist regimen	904 (47.06)	166 (46.11)	732 (46.24)	
Microflare protocol	100 (5.21)	11 (3.06)	85 (5.37)	
Others	20 (1.04)	2 (0.56)	23 (1.45)	
**Type of insemination**				
IVF	1276 (66.42)	240 (66.67)	1063 (67.15)	0.901
ICSI	645 (33.58)	120 (33.33)	520 (32.85)	
**Number of oocytes retrieved**				
≤ 10	712 (37.06)	123 (34.17)	535 (33.80)	0.171
11–20	873 (45.45)	161 (44.72)	739 (46.68)	
> 20	336 (17.49)	76 (21.11)	309 (19.52)	
**Characteristics of FET cycle**				
**Day of embryo transfer**				
Day3	1049 (73.35)	258 (71.67)	1140 (72.02)	0.724
Day4	299 (15.56)	56 (15.56)	267 (16.87)	
Day5	213 (11.09)	46 (12.78)	176 (11.12)	
**Number of embryo transferred**				
Single embryo transfer	214 (11.14)	33 (9.17)	181 (11.43)	0.811
Multiple embryo transfer	1707 (88.86)	327 (90.83)	1402 (88.57)	
**Hormone level before embryo transfer**				
E2, Median [IQR], ×10^3^ pmol/L	1.20 [0.86–1.88]	1.64 [1.01–2.69]	1.86 [1.07–3.22]	< 0.001
P4, Median [IQR], nmol/L	2.90 [1.80–3.60]	2.50 [1.50–2.60]	1.50 [0.90–2.40]	< 0.001
LH, Median [IQR], IU/L	14.05 [9.20–24.90]	10.60 [6.90–17.40]	12.00 [7.50–20.30]	< 0.001
**Endometrial thickness, Mean ± SD, mm**	9.54 ± 1.46	9.52 ± 1.52	9.55 ± 1.43	0.952

*FET* frozen embryo transfer, *NC* natural cycles, *OS* ovarian stimulation, *HRT* hormonal replacement therapy, *COH* controlled ovarian stimulation, *IVF* in vitro fertilization, *ICSI* intracytoplasmic sperm injection, *E2* estradiol, *P4* progesterone, *LH* luteinizing hormone

The results of the multivariable analysis of pregnancy-related complications among all pregnant women is shown in Table 3. For both singleton and multiple deliveries, the risk of preeclampsia was higher when the endometrium was prepared with an OS (singleton delivery: aOR = 2.24, 95%CI: 1.48–3.38; multiple delivery: aOR = 2.91, 95%CI: 1.56–5.41) or HRT protocol (singleton delivery: aOR = 1.88, 95%CI: 1.43–2.48; multiple delivery: aOR = 2.43, 95%CI: 1.56–3.78) than when an NC protocol was used. Despite the null effect found between different endometrial preparation protocols and the risk of gestational hypertension among singleton deliveries, HRT cycles were found to increase the risk of gestational hypertension among multiple deliveries (aOR = 1.99, 95%CI: 1.19–3.32). Furthermore, preparing the endometrium with an OS or HRT protocol also increased the risk of ICP (OS protocol: singleton delivery: aOR = 2.09, 95%CI: 1.21–3.59, multiple delivery: aOR = 2.62, 95%CI: 1.07–6.38; HRT protocol: singleton delivery: aOR = 1.85, 95%CI: 1.29–2.63, multiple delivery: aOR = 2.37, 95%CI: 1.25–4.48). A positive association was found between meconium staining of the amniotic fluid and the use of HRT cycles in singleton deliveries (aOR = 1.31, 95%CI: 1.06–1.61), while no such association was observed for multiple deliveries (aOR = 1.07, 95%CI: 0.70–1.63). Additionally, we did not find any associations between the different endometrial preparation protocols and other pregnancy-related complications, including GDM, preterm birth, and cesarean section, for either singleton or multiple deliveries.

**Table 3:** Pregnancy-related complications of pregnancies carried to delivery following FET with different endometrial preparation protocols

	Singleton delivery					Multiple delivery				
	NC (***N*** = 1491)	OS (***N*** = 272)	HRT (***N*** = 1234)	aOR (95%CI)^**a**^	aOR (95%CI)^**a**^	NC (***N*** = 430)	OS (***N*** = 88)	HRT (***N*** = 349)	aOR (95%CI)^**a**^	aOR (95%CI)^**a**^
	No. (%)	No. (%)	No. (%)	OS vs.NC	HRT vs. NC	No. (%)	No. (%)	No. (%)	OS vs.NC	HRT vs. NC
**Gestational diabetes mellitus**										
No	1351 (90.61)	237 (87.13)	1110 (89.95)	Reference	Reference	383 (89.07)	75 (85.23)	323 (92.82)	Reference	Reference
Yes	140 (9.39)	35 (12.87)	124 (10.05)	1.44 (0.97–2.16)	1.07 (0.82–1.40)	47 (10.93)	13 (14.77)	25 (7.18)	1.38 (0.70–2.71)	0.63 (0.37–1.06)
**Hypertensive disorder**										
No	1301 (87.26)	216 (79.41)	998 (80.88)	Reference	Reference	361 (83.95)	66 (75.00)	243 (69.63)	Reference	Reference
Gestational hypertension	91 (6.10)	20 (7.35)	90 (7.29)	1.34 (0.80–2.26)	1.31 (0.96–1.79)	30 (6.98)	7 (7.95)	41 (11.75)	1.38 (0.58–3.32)	1.99 (1.19–3.32)
Preeclampsia	99 (6.64)	36 (13.24)	146 (11.83)	2.24 (1.48–3.38)	1.88 (1.43–2.48)	39 (9.07)	19 (21.59)	65 (18.62)	2.91 (1.56–5.41)	2.43 (1.56–3.78)
**Intrahepatic cholestasis of pregnancy**										
No	1433 (96.11)	253 (93.01)	1152 (93.35)	Reference	Reference	414 (96.28)	80 (90.91)	319 (91.40)	Reference	Reference
Yes	58 (3.89)	19 (6.99)	82 (6.65)	2.09 (1.21–3.59)	1.85 (1.29–2.63)	16 (3.72)	8 (9.09)	30 (8.60)	2.62 (1.07–6.38)	2.37 (1.25–4.48)
**Meconium staining of the amniotic fluid**										
No	1274 (85.45)	225 (82.72)	1012 (82.01)	Reference	Reference	372 (86.51)	76 (86.36)	299 (85.67)	Reference	Reference
Yes	217 (14.55)	47 (17.28)	222 (17.99)	1.27 (0.90–1.80)	1.31 (1.06–1.61)	58 (13.49)	12 (13.64)	50 (14.33)	1.07 (0.54–2.11)	1.07 (0.70–1.63)
**Preterm birth**										
No	1310 (87.86)	251 (92.28)	1064 (86.22)	Reference	Reference	210 (48.84)	38 (43.18)	150 (42.98)	Reference	Reference
Preterm	181 (12.14)	21 (7.72)	170 (13.78)	0.63 (0.39–1.02)	1.21 (0.95–1.53)	220 (51.16)	50 (56.82)	199 (57.02)	1.32 (0.82–2.11)	1.27 (0.95–1.70)
**Mode of delivery**										
Vaginal	665 (44.60)	109 (40.07)	480 (38.90)	Reference	Reference	17 (3.95)	4 (4.55)	13 (3.72)	Reference	Reference
Cesarean section	826 (55.40)	163 (59.93)	754 (61.10)	1.15 (0.88–1.50)	1.24 (0.96–1.45)	413 (96.05)	84 (95.45)	336 (96.28)	0.93 (0.30–2.88)	1.15 (0.54–2.47)

*FET* frozen embryo transfer, *NC* natural cycles, *OS* ovarian stimulation, *HRT* hormonal replacement therapy, *aOR* adjusted odds ratio, *CI* confidence interval

^a^aOR was adjusted for age at oocyte retrieval, age at embryo transfer, BMI and cause of infertility

Table 4 indicates the associations between different endometrial preparation protocols and neonatal birth weight. Preparing the endometrium with different protocols resulted in similar gender proportions in neonates in both singletons and multiples. The risk of LBW was significantly higher for the OS protocol in both singleton and multiple deliveries (singletons: aOR = 1.71, 95%CI: 1.02–2.87; multiples: aOR = 1.43, 95%CI: 1.03–1.99), and the same effect was observed for HRT protocols (singletons: aOR = 1.93, 95%CI: 1.39–2.68; multiples: aOR = 1.69, 95%CI: 1.37–2.08). Furthermore, preparing the endometrium with the HRT protocol increased the risk of SGA in both singletons (aOR = 1.27, 95%CI: 1.05–1.70) and multiples (aOR = 2.33, 95%CI: 1.79–3.03), and the OS protocol increased the risk of SGA in singletons (aOR = 1.58, 95%CI: 1.02–2.43), but not multiples (aOR = 1.39, 95%CI: 0.91–2.13). Additionally, there was no evidence of an association between LGA or macrosomia and endometrial preparation protocols in either singletons or multiples.

**Table 4:** Outcomes of neonates born following FET with different endometrial preparation protocols

	Singletons delivery					Multiple delivery				
	NC (***N*** = 1491)	OS (***N*** = 272)	HRT (***N*** = 1234)	aOR (95%CI) ^**a**^	aOR (95%CI) ^**a**^	NC (***N*** = 861)	OS (***N*** = 176)	HRT (***N*** = 698)	aOR (95%CI) ^**a**^	aOR (95%CI) ^**a**^
	No. (%)	No. (%)	No. (%)	OS vs.NC	HRT vs. NC	No. (%)	No. (%)	No. (%)	OS vs.NC	HRT vs. NC
**Gender**										
Male	733 (49.16)	149 (54.78)	625 (50.65)	Reference	Reference	429 (49.83)	80 (45.45)	373 (53.44)	Reference	Reference
Female	758 (50.84)	123 (45.22)	609 (49.35)	0.80 (0.62–1.04)	0.94 (0.80–1.10)	432 (50.17)	96 (54.55)	325 (46.56)	1.24 (0.89–1.73)	0.89 (0.73–1.10)
**Birthweight**										
< 2500 g	68 (4.56)	23 (8.46)	112 (9.08)	1.71 (1.02–2.87)	1.93 (1.39–2.68)	368 (42.74)	90 (51.14)	386 (55.30)	1.43 (1.03–1.99)	1.69 (1.37–2.08)
2500-4000 g	1319 (88.46)	226 (83.09)	1031 (83.55)	Reference	Reference	493 (57.26)	86 (48.86)	312 (44.70)	Reference	Reference
> 4000 g	104 (6.98)	23 (8.46)	91 (7.37)	1.19 (0.74–1.91)	1.01 (0.84–1.36)	0 (0.00)	0 (0.00)	0 (0.00)	NA	NA
**Birthweight for gestational age**										
SGA	104 (6.98)	32 (11.76)	126 (10.21)	1.58 (1.02–2.43)	1.27 (1.05–1.70)	127 (14.75)	35 (19.89)	203 (29.08)	1.39 (0.91–2.13)	2.33 (1.79–3.03)
AGA	1037 (69.55)	189 (69.49)	857 (69.45)	Reference	Reference	689 (80.02)	136 (77.27)	464 (66.48)	Reference	Reference
LGA	350 (23.47)	51 (18.75)	251 (20.34)	0.74 (0.52–1.03)	0.82 (0.67–1.01)	45 (5.23)	5 (2.84)	31 (4.44)	0.45 (0.16–1.21)	0.92 (0.55–1.54)

*FET* frozen embryo transfer, *NC* natural cycles, *OS* ovarian stimulation, *HRT* hormonal replacement therapy, *aOR* adjusted odds ratio, *CI* confidence interval, *NA* not accessible, *AGA* appropriate for gestational age, *SGA* small for gestational age, *LGA* large for gestational age

^a^aOR was adjusted for age at oocyte retrieval, age at embryo transfer, BMI, cause of infertility, gestational hypertensive disorder, intrahepatic cholestasis of pregnancy, and meconium staining of the amniotic fluid

We further restricted the analysis to singleton cases and stratified the analysis of pregnancy-related complications (Table S1) and neonatal birth weight (Table S2) according to the number of embryo transfers. In women with single embryo transfer, the risk of preeclampsia, ICP, LBW and SGA were partially eliminated when the endometrium was prepared with an OS or HRT protocol. However, pregnant women who underwent double embryo transfer were still at risk of preeclampsia, ICP, LBW and SGA after an OS or HRT protocol. Additionally, these findings were also confirmed by interactive models that number of FET have interactive effects with OS or HRT protocol on increased risk of preeclampsia, ICP, and LBW (Table S3–4).

## Discussion

In this retrospective cohort of 12,950 FET cycles, we found that the live birth rate after FET was slightly higher and the early pregnancy loss rate significantly lower in patients in whom the endometrium was prepared with an NC protocol than when HRT or OS protocols were used. In addition, among patients with live births, compared to HRT or OS protocols, preparing the endometrium with the NC protocol were found to be associated with the decreased risk of pregnancy-related complications, including gestational hypertension, preeclampsia, and ICP, as well as the decreased risk of LBW and SGA.

The approach used to prepare the endometrium for FET is often personalized for patients. Commonly, natural cycles are only applied to ovulatory women with regular menstruation, while HRT protocols are performed in all women regardless of menstrual regularity and offers flexibility for FET [5]. Another endometrial preparation protocol of ovarian stimulation is also used to improve certain defects in the follicular and subsequent luteal phase, thus enhancing endometrial receptivity [18]. This is the reason for our findings indicating a higher proportion of women with anovulatory in OS or HRT cycle groups than in the NC groups. Furthermore, the most critical discrepancy among different endometrial preparation protocols is the alteration of maternal hormone levels. Unlike natural cycles, in which the endocrine preparation of the endometrium is achieved by endogenous hormones from a developing follicle, in HRT protocols, the endometrium is prepared with exogenous estradiol and progesterone, while in OS protocols, the follicle cohort is induced with exogenous gonadotropins or an aromatase inhibitor [18, 19]. It has been demonstrated that both estradiol and progesterone levels are aberrant in either HRT or OS protocols [9, 11]. Although FET is thought to prevent patients from experiencing the superphysiological hormonal milieu observed in fresh embryo transfer, preparing the endometrium with HRT or OS protocols still leads to alterations in maternal hormone levels. Thus, in our study, serum estradiol levels were elevated after the endometrium was prepared with HRT or OS protocols. The levels of LH and progesterone were significantly lower in the HRT group than in NC group due to the suppression of normal regulation.

Different endometrial preparation regimens are used to obtain optimal conditions for embryo implantation following FET. However, there is no conclusive evidence indicating that one of these approaches is superior to another. A meta-analysis including 7 retrospective studies and 1 randomized controlled trial revealed that comparable results with regard to the clinical pregnancy rate, ongoing pregnancy rate, and livebirth rate were achieved between NC and HRT protocols [20]. Additionally, another meta-analysis failed to find any superiority between the effects of NC and OS protocols on the pregnancy rate [5]. Based on our findings, although these three protocols produced similar rates of chemical pregnancy, clinical pregnancy, and ongoing pregnancy, we found that the HRT protocol achieved a lower rate of live birth than was obtained using the NC and OS protocols. Furthermore, the chance of early pregnancy loss was also higher for OS and HRT protocols. Ezoe et al. and Horcajadas et al. reported that decidualization might be impaired, leading to a subsequent decrease in endometrial receptivity after ovarian stimulation [11, 21]. This might help to explain the increased rate of early pregnancy loss observed after OS protocols. However, a possible reason for the higher rate of early pregnancy loss observed in HRT cycles might be the excessive estradiol in the environment or a suboptimal progesterone to estradiol ratio [7, 22].

Compared to the attention around regarding the effects of different endometrial preparation protocols on pregnancy outcomes following FET, pregnancy-related complications are rarely considered. Recently, Saito et al. first reported that preparing the endometrium with HRT was associated with higher risks of hypertensive disorders and placenta accrete and a lower risk of GDM [23]. In contrast to Saito et al., who excluded the OS protocol, our study included all FET cycles with OS regimens despite their limited number. Our study adds some interesting new findings to the literature following on Saito et al., in which these cycles were not mentioned. In addition to hypertensive disorders, including gestational hypertension and preeclampsia, we also found that the risk of ICP was higher when using both OS and HRT protocols than when using the NC protocol. However, we did not find any association between GDM and OS or HRT protocols. Our previous study indicated that COH-induced high estradiol levels are sustained for more than 8 weeks following fresh embryo transfer and can lead to adverse effects on endometrial receptivity and intrauterine fetal growth [24]. Although FET could effectively protect patients from the adverse effects of high maternal E2 during early pregnancy, estrogen supplementation during the period of HRT and OS-induced higher estradiol levels would still affect maternal serum estradiol levels to some extent. In vivo studies performed in nonhuman primates have indicated that activated E2 signaling might be involved in regulating trophoblast differentiation in early pregnancy and subsequently results in the insufficient invasion of the trophoblast and utero-placental vessel remodeling [25, 26]. Impaired utero-placental blood flow might influence intrauterine fetal growth and several obstetric outcomes, including preeclampsia and SGA [27]. Thus, it is not surprising to find that the risk of LBW and SGA was higher following the OS and HRT protocols in our study. Importantly, multiple pregnancy is one of the critical risk factors for maternal and fetal comorbidities including gestational hypertensive disorders, LBW and SGA [28, 29], thus that is why the incidence of gestational hypertensive disorders, LBW and SGA reported higher in multiple deliveries stratification in our study. In addition, elevated estradiol level was also regarded to be negatively associated with bile salt export pump, which is responsible for biliary secretion of bile acids, thus subsequently suppressed the enterohepatic circulation of bile acids and contributed to the ICP [30].

Although preparing the endometrium with NC provides patients with many benefits with regard to perinatal health, the OS and HRT protocol still have advantages in terms of minimal monitoring and easy scheduling with FET procedures and are especially helpful to patients with irregular menstrual cycles or who are anovulatory. If an OS or HRT protocol is required in the process of FET, we will recommend a single embryo transfer rather than double embryo transfer according to our findings shown in Tables S1 and S2. Single embryo transfer could partially minimize the risk of adverse effects on pregnancy-related complications and neonatal birth weight induced by OS and HRT protocol. A possible explanation of these findings might be the increased chance that embryos will implant on unfavorable sites during multiple embryo transfer than during single embryo transfer. Animal experiments have indicated that embryos implanted at unfavorable sites are associated with pregnancy complications [30].

As this is a hospital-based retrospective cohort study, we failed to rule out some unknown confounders that might influence the risk of obstetric complications and newborn birth weight; these included nutrient intake and physical activities performed during pregnancy. For instance, vitamin D was believed to improve the pregnancy outcomes after embryo transfer [31, 32], and some of patients administrates vitamin D to modulate reproductive outcomes. However, as vitamin D could be available over-the-counter, we failed to record the details of vitamin D supplementation, which might cause bias in this analysis. Limited sample size is another limitation in this study, especially for multiple pregnancy stratification, it reduced statistical power when analyzing maternal and neonatal outcomes. Furthermore, bias induced by patients’ favoring of different endometrial preparation protocols is another inevitable limitation due to the retrospective design of this study. In addition, hormone levels were measured before FET at a time equivalent to the day of oocyte retrieval, when progesterone supplementation was not yet started. Thus, these findings may not reflect the actual level of progesterone after HRT, and we failed to confirm any associations between the level of progesterone after HRT and any pregnancy outcomes, pregnancy-related complications and neonatal outcomes. Although our study is not the first to reveal that HRT protocols affect obstetric complications, our findings add new information to what is known about the association between OS protocols and obstetric complications and neonatal birth weight. Moreover, our stratified analysis confirms the benefit of single embryo transfer on OS- or HRT-induced adverse effects with regard to complications and neonatal birth weight. Ideally, future randomized control trials exploring the issue of endometrial preparation protocols should give more attention to pregnancy-related complications as one of the study outcomes.

## Conclusion

In summary, our findings showed that with the exception of the application of OS or HRT protocols in women who are anovulatory, preparing the endometrium with natural cycles should be encouraged to avoid the aberrant hormone levels observed before FET in women with regular menstrual cycles. Although the NC protocol requires more frequent visits to the hospital and endocrine and ultrasonographic monitoring, it also helps women to optimize pregnancy outcomes and improve maternal health during pregnancy. Caution should be warranted when using OS or HRT protocols, due to the associations with gestational hypertension, preeclampsia, and ICP as well as LBW and SGA.

## Supplementary Information

**Additional file 1: Table S1.** Pregnancy-related complications of singleton deliveries following FET with different endometrial preparation protocols stratified according to the number of embryo transfer. **Table S2.** Outcomes of singletons born following FET with different endometrial preparation protocols stratified according to the number of embryo transfer. **Table S3.** Interactive effect of number of FET with different endometrial preparation protocols on pregnancy-related complications of singleton deliveries following FET. **Table S4.** Interactive effect of number of FET with different endometrial preparation protocols on outcomes of singletons born following FET

## Data Availability

The data can be available from the corresponding author on the resealable request.

## Abbreviations

ART: Assisted reproductive technology
BMI: Body mass index
CI: Confidence intervals
COH: Controlled ovarian hyperstimulation
FET: Frozen-thawed embryo transfer
GDM: Gestational diabetes mellitus
HRT: Hormone replacement therapy
ICP: Intrahepatic cholestasis of pregnancy
ICSI: Intracytoplasmic sperm injection
IVF: In vitro fertilization
LBW: Low birth weight
LGA: Large for gestational age
LH: Luteinizing hormone
NC: Natural cycles
OR: Odds ratios
OS: Ovarian stimulation
PGT: Preimplantation genetic testing
SGA: Small for gestational age
