# Supplementary material for: Perinatal outcomes of neonates born from different endometrial preparation protocols after frozen embryo transfer: a retrospective cohort study
Source: BMC Pregnancy Childbirth. 2021 Apr 29;21:341. doi: 10.1186/s12884-021-03791-9 (PMC8086106; doi:10.1186/s12884-021-03791-9)
Supplement: Supplementary file 1 — Additional file 1: Table S1. Pregnancy-related complications of singleton deliveries following FET with different endometrial preparation protocols stratified according to the number of embryo transfer. Table S2. Outcomes of singletons born following FET with different endometrial preparation protocols stratified according to the number of embryo transfer. Table S3. Interactive effect of number of FET with different endometrial preparation protocols on pregnancy-related complications of singleton deliveries following FET. Table S4. Interactive effect of number of FET with different endometrial preparation protocols on outcomes of singletons born following FET [file 12884_2021_3791_MOESM1_ESM.pdf]

Supplementary Materials

Perinatal outcomes of neonates born from different endometrial preparation protocols after frozen embryo transfer: a retrospective cohort study

Short title: endometrial preparation and perinatal outcomes

Cheng Li[1,2,†], Yi-Chen He[1,2,†], Jing-Jing Xu[1,2,†], Yu Wang[1,2], Han Liu[1,2], Chen-Chi Duan[1,2], Chao-Yi Shi[1,2], Lei Chen[1,2], Jie Wang[1,2], Jian-Zhong Sheng[3], He-Feng Huang[1,2,4*], Yan-Ting Wu[1,2,4*]

[1] International Peace Maternity and Child Health Hospital, School of Medicine, Shanghai Jiao Tong University, Shanghai, 200030, China

[2] Shanghai Key Laboratory of Embryo Original Diseases, Shanghai, 200030, China

[3] Department of Pathology and Pathophysiology, School of Medicine, Zhejiang University, Hangzhou, Zhejiang, China

[4] Obstetrics and Gynecology Hospital, Fudan University, Shanghai, China

[†] Cheng Li, Yi-Chen He, and Jing-Jing Xu contributed equally to this work.

* Correspondence: He-Feng Huang & Yan-Ting Wu, International Peace Maternity and Child Health Hospital, School of Medicine, Shanghai Jiao Tong University, No. 910, Hengshan Rd., Shanghai 200030, China. Obstetrics and Gynecology Hospital, Fudan University, No.419, Fangxie Rd., Shanghai 200000, China

E-mail: yanting_wu@163.com & huanghefg@sjtu.edu.cn.

Table S1 Pregnancy-related complications of singleton deliveries following FET with different endometrial preparation protocols stratified according to the number of embryo transfer

	Single embryo transfer					Multiple embryo transfer				
	NC (N=211)	OS (N=32)	HRT (N=180)	aOR (95%CI) [a]	aOR (95%CI) [a]	NC (N=1280)	OS (N=240)	HRT (N=1054)	aOR (95%CI) [a]	aOR (95%CI) [a]
	No. (%)	No. (%)	No. (%)	OS vs.NC	HRT vs. NC	No. (%)	No. (%)	No. (%)	OS vs.NC	HRT vs. NC
**Gestational diabetes mellitus**										
No	194 (91.94)	28 (87.50)	163 (90.56)	Reference	Reference	1157 (90.39)	209 (87.08)	947 (89.85)	Reference	Reference
Yes	17 (8.06)	4 (12.50)	17 (9.44)	2.02 (0.59-6.90)	1.49 (0.71-3.15)	123 (9.61)	31 (12.92)	107 (10.15)	1.41 (0.92-2.16)	1.04 (0.78-1.38)
**Hypertensive disorder**										
No	190 (90.05)	30 (93.75)	155 (86.11)	Reference	Reference	1111 (86.80)	186 (77.50)	843 (79.98)	Reference	Reference
Gestational hypertension	9 (4.27)	0 (0.00)	13 (7.22)	NA	1.77 (0.71-4.44)	82 (6.41)	20 (8.33)	77 (7.31)	1.48 (0.88-2.49)	1.26 (0.90-1.76)
Preeclampsia	12 (5.69)	2 (6.25)	12 (6.67)	1.20 (0.25-5.79)	1.32 (0.56-3.10)	87 (6.80)	34 (14.17)	134 (12.71)	2.37 (1.54-3.65)	1.99 (1.48-2.67)
**Intrahepatic cholestasis of pregnancy**										
No	204 (96.68)	32 (100.00)	173 (96.11)	Reference	Reference	1229 (96.02)	221 (92.08)	979 (92.88)	Reference	Reference
Yes	7 (3.32)	0 (0.00)	7 (3.89)	NA	1.24 (0.40-3.85)	51 (3.98)	19 (7.92)	75 (7.12)	2.35 (1.35-4.10)	1.94 (1.33-2.84)
**Meconium staining of the amniotic fluid**										
No	175 (82.94)	26 (81.25)	150 (83.33)	Reference	Reference	1099 (85.86)	199 (82.92)	862 (81.78)	Reference	Reference
Yes	36 (17.06)	6 (18.75)	30 (16.67)	1.30 (0.49-3.48)	0.97 (0.56-1.69)	181 (14.14)	41 (17.08)	192 (18.22)	1.29 (0.89-1.88)	1.38 (1.10-1.74)
**Preterm birth**										
No	199 (94.31)	30 (93.75)	164 (91.11)	Reference	Reference	1111 (86.80)	221 (92.08)	900 (85.39)	Reference	Reference
Preterm	12 (5.69)	2 (6.25)	16 (8.89)	1.00 (0.21-4.84)	1.28 (0.55-2.98)	169 (13.20)	19 (7.92)	154 (14.61)	0.60 (0.37-1.00)	1.21 (0.94-1.54)
**Mode of delivery**										
Vaginal	103 (48.82)	15 (46.88)	58 (32.22)	Reference	Reference	562 (43.91)	94 (39.17)	422 (40.04)	Reference	Reference
Cesarean section	108 (51.18)	17 (53.13)	122 (67.78)	1.03 (0.48-2.21)	2.05 (1.33-3.15)	718 (56.09)	146 (60.83)	632 (59.96)	1.16 (0.87-1.54)	1.14 (0.96-1.35)

FET, frozen embryo transfer; NC, natural cycles; OS, ovarian stimulation; HRT, hormonal replacement therapy; aOR, adjusted odds ratio; CI, confidence interval.

a aOR was adjusted for age at oocyte retrieval, age at embryo transfer, BMI and cause of infertility.

Table S2 Outcomes of singletons born following FET with different endometrial preparation protocols stratified according to the number of embryo transfer

	Single embryo transfer					Double embryo transfer				
	NC (N=211)	OS (N=32)	HRT (N=180)	aOR (95%CI) [a]	aOR (95%CI) [a]	NC (N=1280)	OS (N=240)	HRT (N=1054)	aOR (95%CI) [a]	aOR (95%CI) [a]
	No. (%)	No. (%)	No. (%)	OS vs.NC	HRT vs. NC	No. (%)	No. (%)	No. (%)	OS vs.NC	HRT vs. NC
**Gender**										
Male	101 (47.87)	18 (56.25)	86 (47.78)	Reference	Reference	632 (49.38)	131 (54.58)	539 (51.14)	Reference	Reference
Female	110 (52.13)	14 (43.75)	94 (52.22)	0.74 (0.34-1.61)	0.97 (0.64-1.47)	648 (50.63)	109 (45.42)	515 (48.86)	0.82 (0.62-1.08)	0.93 (0.78-1.10)
**Birthweight**										
<2500 g	4 (1.90)	2 (6.25)	8 (4.44)	3.61 (0.60-21.50)	3.30 (0.43-14.78)	64 (5.00)	21 (8.75)	104 (9.87)	1.61 (0.94-2.76)	1.88 (1.33-2.65)
2500-4000g	191 (90.52)	26 (81.25)	158 (87.78)	Reference	Reference	1128 (88.13)	200 (83.33)	873 (82.83)	Reference	Reference
>4000 g	16 (7.58)	4 (12.50)	14 (7.78)	1.80 (0.54-6.05)	1.00 (0.45-2.24)	88 (6.88)	19 (7.92)	77 (7.31)	1.09 (0.65-1.85)	1.01 (0.73-1.41)
**Birthweight for gestational age**										
SGA	16 (7.58)	7 (21.88)	13 (7.22)	4.05 (1.43-12.59)	0.95 (0.44-2.09)	88 (6.88)	25 (10.42)	113 (10.72)	1.35 (1.01-2.19)	1.22 (1.08-1.97)
AGA	152 (72.04)	19 (59.38)	136 (75.56)	Reference	Reference	885 (69.14)	170 (70.83)	721 (68.41)	Reference	Reference
LGA	43 (20.38)	6 (18.75)	31 (17.22)	1.22 (0.45-3.32)	0.77 (0.45-1.32)	307 (23.98)	45 (18.75)	220 (20.87)	0.71 (0.49-1.01)	0.82 (0.66-1.01)

NC, natural cycles; OS, ovarian stimulation; HRT, hormonal replacement therapy; aOR, adjusted odds ratio; CI, confidence interval; NA, not accessible; AGA, appropriate for gestational age; SGA, small for gestational age; LGA, large for gestational age.

a aOR was adjusted for age at oocyte retrieval, age at embryo transfer, BMI, cause of infertility, gestational hypertensive disorder, intrahepatic cholestasis of pregnancy, and meconium staining of the amniotic fluid.

Table S3 Interactive effect of number of FET with different endometrial preparation protocols on pregnancy-related complications of singleton deliveries following FET

	OS vs. NC		HRT vs. NC	
	**aOR (95%CI) [a]**	***P** for interaction*	**aOR (95%CI) [a]**	***P** for interaction*
**Gestational diabetes mellitus**				
No	Reference		Reference	
Yes	1.22 (0.99-1.50)	0.057	1.04 (0.91-1.19)	0.582
**Hypertensive disorder**				
No	Reference		Reference	
Gestational hypertension	1.20 (0.93-1.56)	0.159	1.16 (0.98-1.36)	0.081
Preeclampsia	1.58 (1.28-1.95)	<0.001	1.45 (1.26-1.67)	<0.001
**Intrahepatic cholestasis of pregnancy**				
No	Reference		Reference	
Yes	1.55 (1.17-2.04)	0.002	1.42 (1.19-1.71)	<0.001
**Meconium staining of the amniotic fluid**				
No	Reference		Reference	
Yes	1.11 (0.92-1.33)	0.271	1.15 (1.04-1.29)	0.009
**Preterm birth**				
No	Reference		Reference	
Preterm	0.80 (0.62-1.03)	0.079	1.13 (0.99-1.27)	0.053
**Mode of delivery**				
Vaginal	Reference		Reference	
Cesarean section	1.08 (0.94-1.24)	0.302	1.09 (1.00-1.19)	0.091

FET, frozen embryo transfer; NC, natural cycles; OS, ovarian stimulation; HRT, hormonal replacement therapy; aOR, adjusted odds ratio; CI, confidence interval.

a aOR was adjusted for age at oocyte retrieval, age at embryo transfer, BMI and cause of infertility.

Table S4 Interactive effect of number of FET with different endometrial preparation protocols on outcomes of singletons born following FET

	OS vs. NC		HRT vs. NC	
	**aOR (95%CI) [a]**	***P** for interaction*	**aOR (95%CI) [a]**	***P** for interaction*
**Gender**				
Male	Reference		Reference	
Female	0.89 (0.78-1.02)	0.105	0.96 (0.88-1.04)	0.317
**Birthweight**				
<2500 g	1.32 (1.01-1.73)	0.038	1.45 (1.22-1.72)	<0.001
2500-4000g	Reference		Reference	
>4000 g	1.07 (0.83-1.37)	0.619	0.99 (0.85-1.17)	0.947
**Birthweight for gestational age**				
SGA	1.19 (0.73-1.04)	0.080	1.15 (1.00-1.33)	0.012
AGA	Reference		Reference	
LGA	0.87 (0.73-1.04)	0.093	0.92 (0.83-1.02)	0.085

FET, frozen embryo transfer; NC, natural cycles; OS, ovarian stimulation; HRT, hormonal replacement therapy; aOR, adjusted odds ratio; CI, confidence interval; NA, not accessible; AGA, appropriate for gestational age; SGA, small for gestational age; LGA, large for gestational age.

a aOR was adjusted for age at oocyte retrieval, age at embryo transfer, BMI, cause of infertility, gestational hypertensive disorder, intrahepatic cholestasis of pregnancy, and meconium staining of the amniotic fluid.
